# Dominance in a ground‐dwelling ant community of banana agroecosystem

**DOI:** 10.1002/ece3.2570

**Published:** 2016-11-11

**Authors:** Dominique Carval, Violaine Cotté, Rémi Resmond, Benjamin Perrin, Philippe Tixier

**Affiliations:** ^1^ CIRADUPR GECOLe LamentinMartiniqueFrance; ^2^ CIRADUPR GECOMontpellierFrance; ^3^ Departamento de Agricultura y AgroforesteriaCATIETurrialbaCosta Rica

**Keywords:** pattern analysis, ant, dominance, interference competition, community, coexistence

## Abstract

In tropical ecosystems, ants represent a substantial portion of the animal biomass and contribute to various ecosystem services, including pest regulation and pollination. Dominant ant species are known to determine the structure of ant communities by interfering in the foraging of other ant species. Using bait and pitfall trapping experiments, we performed a pattern analysis at a fine spatial scale of an ant community in a very simplified and homogeneous agroecosystem, that is, a single‐crop banana field in Martinique (French West Indies). We found that the community structure was driven by three dominant species (*Solenopsis geminata*,* Nylanderia guatemalensis*, and *Monomorium ebeninum*) and two subdominant species (*Pheidole fallax* and *Brachymyrmex patagonicus*). Our results showed that dominant and subdominant species generally maintained numerical dominance at baits across time, although *S. geminata*,* M. ebeninum*, and *B. patagonicus* displayed better abilities to maintain dominance than *P. fallax* and *N. guatemalensis*. Almost all interspecific correlations between species abundances, except those between *B. patagonicus* and *N. guatemalensis*, were symmetrically negative, suggesting that interference competition prevails in this ground‐dwelling ant community. However, we observed variations in the diurnal and nocturnal foraging activity and in the daily occurrence at baits, which may mitigate the effect of interference competition through the induction of spatial and temporal niche partitioning. This may explain the coexistence of dominant, subdominant, and subordinate species in this very simplified agroecosystem, limited in habitat structure and diversity.

## 1 Introduction

Ants are ubiquitous, diverse, and abundant and are therefore key components of ecosystems. In tropical ecosystems, ants may represent a substantial portion of the animal biomass (Hölldobler & Wilson, 1990) and may help provide various ecosystem services, including pest regulation and pollination (Perfecto & Vandermeer, 2006; Philpott & Armbrecht, 2006). Hence, an important objective in the study of agroecology is to understand the factors affecting the structure of local ant communities.

The diversity and abundance of ant species and consequently the diversity of ant community structures may be explained by both physiological factors and ecological factors (Philpott & Armbrecht, 2006). The ecological factors can be further divided into habitat‐related factors (e.g., nesting sites, microhabitats, food availability, and food diversity) and ecological interactions (e.g., interspecific competition and foraging interference). Habitat‐related factors strongly influence ant communities through environmental filtering (Wiescher, Pearce‐Duvet, & Feener, 2012), and coexistence of ants in heterogeneous environments has been extensively documented (Dassou, Carval, Depigny, Fansi, & Tixier, 2015; House, Burwell, Brown, & Walters, 2012; Murnen, Gonthier, & Philpott, 2013; Perfecto & Vandermeer, 1996; Vasconcelos, Leite, Vilhena, Lima, & Magnusson, 2008). However, intra‐ and interspecific interactions also play an important role in the structuring of ant communities (Fellers, 1987, 1989). This may be particularly true in homogeneous environments such as agroecosystems.

Intra‐ and interspecific competition can be divided into two types: interference competition, which includes all direct interactions involving aggressive encounters between ants (Fellers, 1987; Kenne et al., 2005), and exploitative competition, when the consumption of a limiting resource by one species reduces the availability of that resource for another species (Fellers, 1987; Kenne et al., 2005). Researchers have hypothesized that the coexistence of ants results from a trade‐off between traits linked to interference competition and those linked to exploitative competition. For example, Fellers (1987) hypothesized that there is a trade‐off between bait discovery and bait dominance, that is, those ant species adept at finding resources have poor interference competitive abilities while those species adept at dominating a resource have good interference competitive abilities but poor resource discovery abilities. However, this trade‐off seems to be the exception rather than the rule (Castracani, Spotti, Grasso, Fanfani, & Mori, 2014; Parr & Gibb, 2012), and positive correlations between discovery and dominance have been reported (Parr & Gibb, 2012).

Interspecific interactions may determine which species are members of particular ant communities. In tropical ant communities, apart from the major arboreal and terrestrial guilds that have specialized foraging habitats, some species forage both in tree canopies and on the ground and may therefore compete (Bluthgen & Feldhaar, 2010). Ant species differ in competitive ability because of differences in foraging activity, colony size, or body size (Hölldobler & Wilson, 1990). In particular, dominant ants can alter the structure of ant communities by interfering in the foraging activity of other ant species (Savolainen & Vepsalainen, 1988). Dominant ants often have mutualistic associations with nonant herbivores that provide honeydew as a sugar source in exchange for protection against predators (Bluthgen, Stork, & Fiedler, 2004). Such mutualisms enable ants to build large colonies with many nests (Richard, Fabre, & Dejean, 2001). Dominant ants achieve superiority because of their aggressiveness, numerical dominance, superior interference behavior, and superior ability to participate in exploitative competition (Parr & Gibb, 2010); such ants are frequently found in disturbed habitats including intensive agroecosystems (King & Tschinkel, 2006). The spatiotemporal dynamics of such dominant ants greatly affect ant community structure (Zakharov, 2002).

In the humid tropics, bananas (*Musa* AAA genome) are mostly grown on bare soil and as a single crop. These semi‐perennial agroecosystems contain regularly spaced banana plants and are extremely simple and homogeneous and, therefore, are well suited for studying ant community structure. In the current study, we performed a pattern analysis at a fine spatial scale and provided information on temporal and spatial dynamics of ants foraging in a single‐crop banana agroecosystem: (i) We assessed the diurnal and nocturnal foraging activity of these species; (ii) determined which species are dominant, subdominant, and subordinate; (iii) assessed how numerical dominance at an impermanent resource (i.e., a bait) evolved through time; and (iv) assessed how abundance of species, at baits and in the neighborhood of the baits, were correlated.

## 2 Materials and Methods

### 2.1 Fields, plots, and subplots

**Figure 1:**
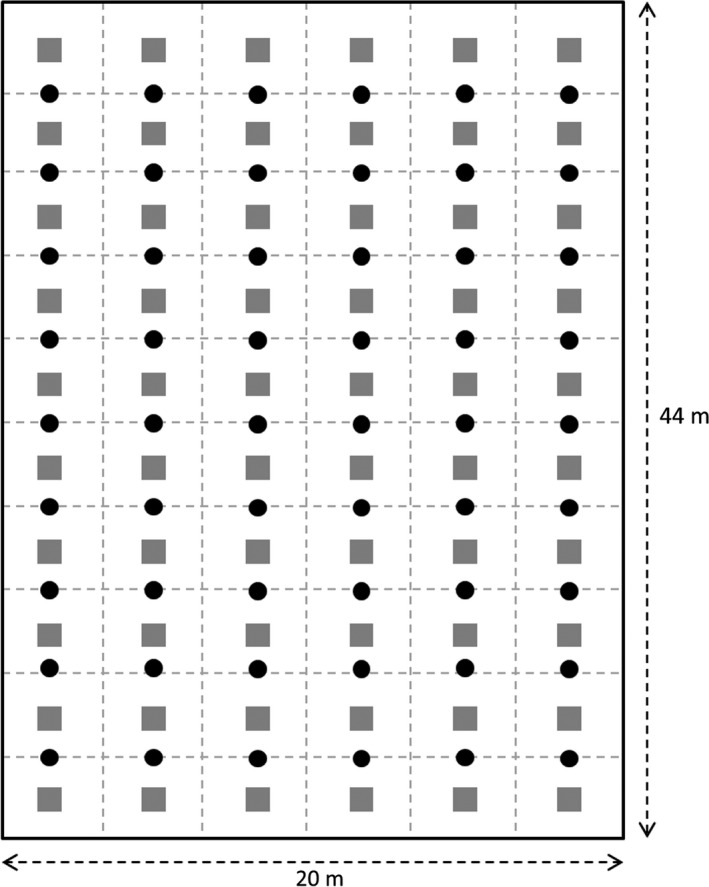
Schema of the experimental design. Gray squares: ceramic tile with baits; black circles: pitfall traps. Subplots correspond to areas delimited by dashed lines

We conducted our study in an experimental banana field (Lamentin, Petit Morne, West French Indies, 14°37′25.1″N, 60°58′07.3″W, 3 m a.s.l) during the dry season (from 23 of April to 19 of June) of 2012. The sampling area or plot was 44 m long and 20 m wide. The banana crop (Cavendish Grande Naine) was in its first cycle when the data were collected to ensure homogeneity across the plot. The age of the banana plantation was 8 months. The climate at the study site is humid tropical with a mean (± *SE*) monthly temperature of 26.5 ± 0.3°C and a mean monthly rainfall of 174.6 ± 21.2 mm. Within the main plot, we defined 60 regularly spaced subplots (14.7 m^2^ each; Figure 1).

### 2.2 Ant sampling: day and night pitfall trapping

To assess the general foraging activity of the ant community, we carried out diurnal and nocturnal pitfall trapping. Pitfall traps were situated between each subplot, with a total of 54 pitfall traps (Figure 1). Pitfall traps contained 50 ml of water with a drop of dishwashing liquid. Pitfall traps were deployed at nightfall at 6:00 p.m., and the trapped ants were collected at 6:00 a.m. and conserved in 70% alcohol for identification. Next, pitfall traps were washed with water and refilled. Trapped ants were then collected at 6.00 p.m. and conserved in 70% alcohol for identification. This sampling method was replicated three times. For day and night period, we calculated the proportion of pitfall traps where the species were recorded as well as their mean abundance per pitfall trap. Next, we used Kruskal–Wallis tests to analyze the day–night variation in foraging activity.

### 2.3 Ant sampling: baiting

One 30 × 30 cm white ceramic tile was placed at the center of each subplot, such that the tiles were regularly spaced across the main plot. In each subplot, we measured ant abundance using canned tuna–honey baits. One bait, which had a diameter of 4 cm, was placed in the center of each ceramic tile on the 23 of April 2012, which we called hereafter date 1. Each subplot was sampled 30, 90, and 180 min (sampling times 1, 2, and 3, respectively) after the baits were deployed. At each sampling time, we identified and counted the individuals of different species present on the tile. The ants were also recorded according to a six‐point abundance scale (following Andersen, 1997; Baccaro, Ketelhut, & De Morais, 2010; Parr, Sinclair, Andersen, Gaston, & Chown, 2005). We used the percentage of bait controlled as a measure of dominance (following Bestelmeyer, 2000; Baccaro et al., 2010) rather than strict bait monopolization (Andersen, 1992). Baits were considered controlled by a species (i) if the number of individuals is >20 and no other ant was present (monopolization) or (ii) if one species was at least twice as numerous as the second numerous taxon when several species were present and the total number of individuals was >20). Samples of all species were collected and conserved in 70% alcohol, then we performed identification to genus according to the Bolton key (Bolton, 1994), and all ants were sent to J. Delabie (Laboratory of Myrmecology, UESC/CEPLAC, Brazil), who identified the ants to species. Sampling was performed between 8:00 and 11:00 in the morning and between 2:00 and 5:00 in the afternoon and repeated the 12 of May 2012, the 31 of May 2012, and the 19 of June 2012, which we called, respectively, hereafter dates 2, 3, and 4. For each species, we used Kruskal–Wallis tests to analyze the daily variation (morning vs. afternoon) in abundances.

### 2.4 Dominant analysis

Following Baccaro et al. (2010), we used a combination of numerical and behavioral criteria of dominance to determine dominant, subdominant, and subordinate ants. An ant species was considered as a dominant species when (i) it occurred at a large proportion of baits; (ii) it controlled a large proportion of baits whenever it was present; and (iii) it had a high mean abundance score (Andersen, 1992; Baccaro et al., 2010; Parr, 2008). The dominant (respectively, subdominant) species were classified as those that were recorded in >10% of all baits, controlled >25% (respectively, >10%) of baits where they occurred, and with a mean abundance score (calculated by dividing the sum of the abundance scores for the species at all baits by the number of baits at which the species was present) of >3 (respectively, >2.5). All other species that did not meet all these criteria were considered as subordinate species.

### 2.5 Statistical analysis

#### 2.5.1 Data mining and probability matrices of transition

We first performed a principal component analysis and a hierarchical cluster analysis, based on Ward's method (Ward, 1963), to classify the observations at baits according to their similarities in species distribution and abundance. We thus obtained a hierarchical classification of observations for each replicate tile at one sampling time. Once the partition of observations into groups on the basis of the Ward's minimum variance agglomerative clustering was obtained, we used that partition as the initial value for K‐means partitioning (Murtagh & Legendre, 2014). This procedure was used to identify the type of “momentary” communities that we observed at baits and to established transition matrices. Moreover, the type of “momentary” community (which we called hereafter a group) observed at sampling time 1 are likely to be indicative of the colonies that was spatially close to the subplot (see below in *Subplot scale dynamics and correlations between ant species abundances*).

Once groups of observations were identified, we named these groups according to the numerically dominant species of each group. We next used the Markov chain approach to define the probability (in the form of probability matrices) that a numerically dominant species will maintain its numerical dominance on the resource or lose it to the benefit of another dominant or subdominant species from sampling time 1 to sampling time 2 and from sampling time 2 to sampling time 3. We then used 10,000 Markov chain simulations to obtain probabilities that each species will maintain numerical dominance from sampling time 1 to sampling time 3. Probability matrices of transition were obtained using the “markovchain” R‐package (Spedicato, 2015).

#### 2.5.2 Autocorrelation

It is important to consider autocorrelation in studies of species interactions (Dormann et al., 2007), and the omission of spatial autocorrelation in analyses may lead to false conclusions (Kuehn, 2007). For the abundance of each of the identified dominant and subdominant species, we tested the spatial autocorrelation using the C index of Geary (1954). Low values of this index indicate that two locations are positively correlated, that is, that they are more likely to resemble each other. High values of this index indicate the absence of correlation between two locations. The C index of Geary was calculated for the abundance of each dominant and subdominant species for each replicate tile at each sampling time and for distances *d* ranging from 2.5 to 6.0 m. The effects of distance on the Geary *C* values were assessed with linear models (LMs) that took the form of *C* = α + β_1_ *d* + β_2_ *d* ^2^ + ε where *α* was the model intercept, β were regression coefficients, and ε was the normally distributed error term.

#### 2.5.3 Subplot scale dynamics and correlations between ant species abundances

We used Poisson generalized mixed‐effect models (GLMMs) to assess correlations between abundances of dominant and subdominant species, which may reflect the outcome of species interactions at baits. Following Zuur, Ieno, Saveliev, and Smith (2009), we first tested for collinearity (i.e., correlation) between covariates using the variance inflation factor (VIF) method. Once the set of explanatory variables (fixed effects) was determined, we tested the random effect structure. Here, for each species *i*, we defined as the fixed effect, the local abundances of species *j* (i.e., abundances in subplots), the mean abundances of conspecifics of *i* and of species *j* in the neighborhood (i.e., abundances in the surrounding subplots situate at a distance defined for each species by the spatial autocorrelation analysis) of the considered subplot, and the sampling time.

Humidity, temperature, and other factors may cause the abundance of a species to be similar between subplots at the same sampling time on one date. To consider the nonindependence between data from subplots at the same sampling time on one date, we introduced this latter variable as a random intercept effect. Moreover, the ants observed at the sampling time 1 are likely to be indicative of the community that was spatially close to the subplot and the data from the three sampling times at a specific subplot are likely to be correlated. Therefore, to consider the nonindependence between sampling times at a same subplot, we introduced the group at sampling time 1, obtained by the data mining procedure, for each subplot as a random slope effect on sampling time. Following Zuur et al. (2009), we tested random effect structures by comparing nested GLMMs comprising all fixed effects. We used Akaike information criteria (AIC) and likelihood ratio tests (LRTs) to select the best random effect structure of the model for each ant species (Bolker et al., 2009).

After using the GLMMs to determine the best random structure for each species, we selected the best model by removing nonsignificant fixed‐effect parameters in a backwards‐stepwise process using LRTs. The selection procedure was continued until a model was found in which all effects were significant (Zuur et al., 2009).

All LMs and GLMMs were estimated using the “glmer” function in the “lme4” package (Bates, Maechler, & Bolker, 2012), in which the maximum likelihood of parameters is approximated by the Laplace method (Bolker et al., 2009). All statistical analyses were performed with R 2.15.0 (R Development Core Team, 2014) and with an alpha level of 0.05.

## 3 Results

### 3.1 Diurnal and nocturnal foraging activity

**Figure A1:**
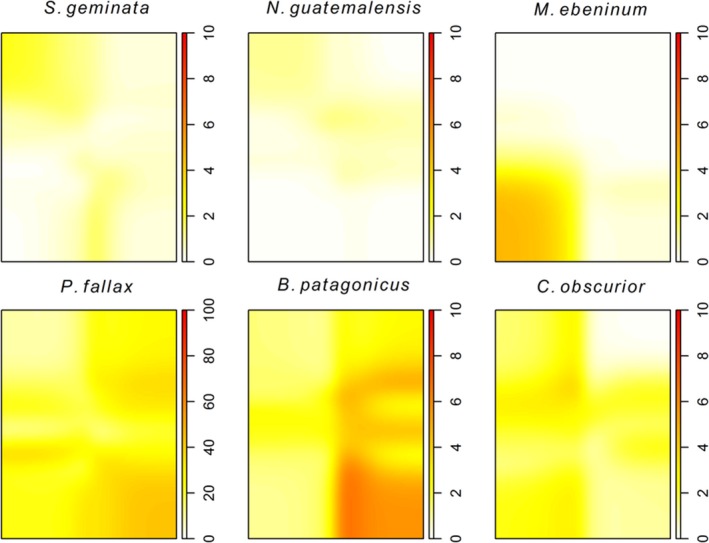
Spatial distribution of the dominant and subdominant ant species and of *Cardiocondyla obscurior* based on abundances recorded in pitfall traps

A total of 6,229 ants belonging to 11 species were collected with pitfall traps. Among the 11 species trapped in pitfall traps, we found only two specimens of *Tetramorium bicarinatum* (Nylander), which species was not included in the analysis. *Solenopsis geminata*,* Nylanderia guatemalensis*,* Monomorium ebeninum*,* Pheidole fallax*, and *Cardiocondyla obscurior* were the most frequently recorded species, while *Wasmannia auropunctata*,* Odontomachus brunneus*,* Camponotus sexguttatus*, and *Paratrechina longicornis* occurred at low frequency (Table 1). *P. fallax* was largely the most frequently trapped species but also the most abundant trapped species in pitfall traps (Table 1). The foraging activity (the percentage of recorded pitfall traps and the mean abundance) of *S. geminata*,* M. ebeninum*,* P. fallax*,* Brachymyrmex patagonicus*,* C. obscurior*, and *P. longicornis* was greater during the day than at night, while *N. guatemalensis* was more active at night than during the day (Table 2). The foraging activity of *W. auropunctata*,* O. brunneus*, and *C. sexguttatus* was similar during the day and at night (Table 2). Maps of spatial distribution of species are provided in the Appendix (Figure A1).

**Table 1:** Occurrence of dominant, subdominant, and subordinate ants at pitfall traps

	Recorded pitfall traps (%)		Mean abundance (95% CI)	
	Day	Night	Day	Night
Dominant				
*Solenopsis geminata* (Fabricius)	29.63	15.43	0.69 (0.46–0.91)	0.21 (0.12–0.30)
*Nylanderia guatemalensis* (Forel)	12.35	33.95	0.14 (0.08–0.21)	0.56 (0.38–0.73)
*Monomorium ebeninum* Forel	22.84	8.02	0.62 (0.40–0.85)	0.13 (0.05–0.21)
Subdominant				
*Pheidole fallax* Mayr	96.91	87.65	23.21 (20.14–26.28)	5.68 (4.44–6.91)
*Brachymyrmex patagonicus* Mayr	67.90	38.27	3.13 (2.64–3.61)	1.11 (0.87–1.35)
Subordinate				
*Cardiocondyla obscurior* Wheeler	81.48	48.77	1.96 (1.60–2.32)	0.58 (0.44–0.72)
*Wasmannia auropunctata* (Roger)	2.47	3.70	0.02 (0.00–0.05)	0.04 (0.01–0.07)
*Odontomachus brunneus* (Patton)	5.56	5.56	0.06 (0.02–0.09)	0.06 (0.02–0.10)
*Camponotus sexguttatus* (Fabricius)	3.70	4.32	0.05 (0.01–0.09)	0.04 (0.01–0.07)
*Paratrechina longicornis* (Latreille)	7.41	2.47	0.09 (0.04–0.14)	0.07 (−0.03 to 0.17)

**Table 2:** Comparisons of the diurnal and nocturnal foraging activity of the dominant, subdominant, and subordinate species (Kruskal–Wallis tests)

	Recorded pitfall traps			Mean abundance		
	Day vs. night			Day vs. night		
	χ^2^	*df*	*p*	χ^2^	*df*	*p*
Dominant						
*Solenopsis geminata*	9.3	1	**.002**	11.1	1	**<.001**
*Nylanderia guatemalensis*	21.2	1	**<.001**	22.6	1	**<.001**
*Monomorium ebeninum*	13.6	1	**<.001**	14.8	1	**<.001**
Subdominant						
*Pheidole fallax*	9.7	1	**.002**	119.7	1	**<.001**
*Brachymyrmex patagonicus*	38.0	1	**<.001**	57.3	1	**<.001**
Subordinate						
*Cardiocondyla obscurior*	28.5	1	**<.001**	42.2	1	**<.001**
*Wasmannia auropunctata*	0.4	1	.521	0.4	1	.521
*Odontomachus brunneus*	0.0	1	1.000	0.0	1	.989
*Camponotus sexguttatus*	0.8	1	.777	0.1	1	.796
*Paratrechina longicornis*	4.2	1	**.040**	4.1	1	**.041**

### 3.2 Dominant, subdominant, and subordinate species

**Figure A2:**
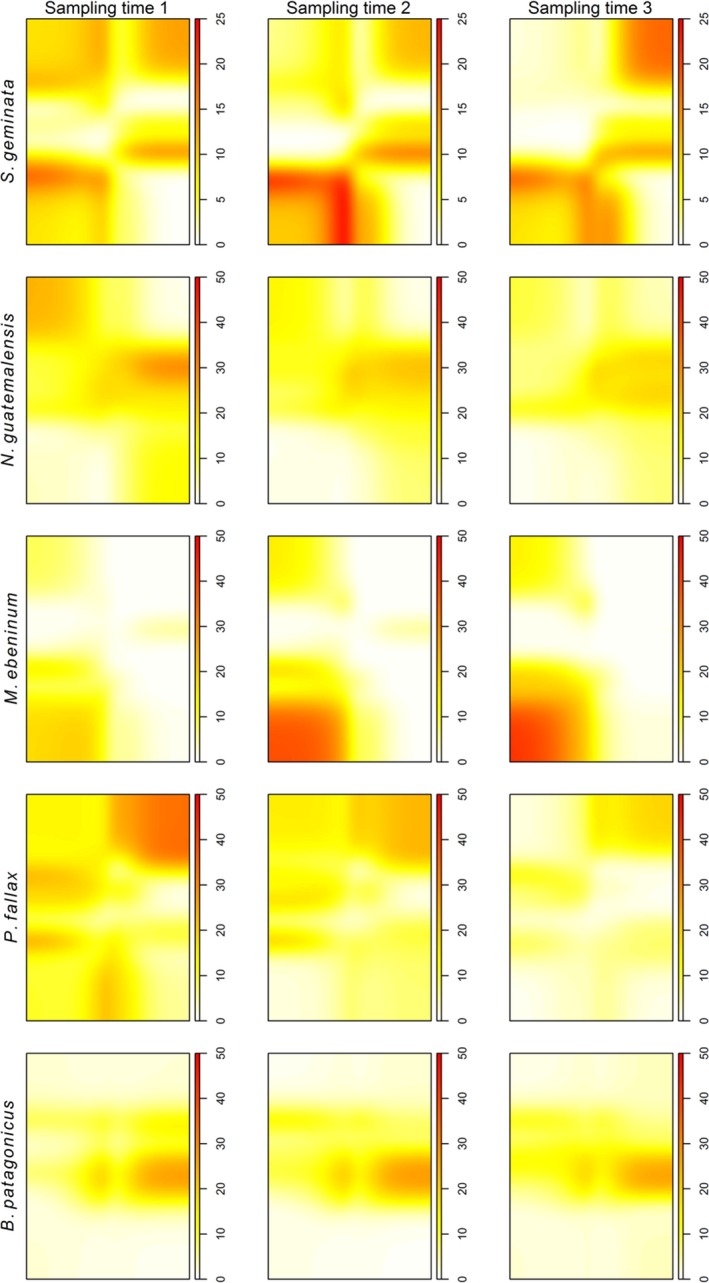
Spatial distribution of the dominant and subdominant ant species based on abundances recorded at baits

A total of 10 species were recorded during bait experiments. These species were the same as recorded in pitfall traps with the exception of *T. bicarinatum*, which was never recorded at baits. At baits, the presence of all species was in relatively high proportion, ranging from 10% to 77% (Table 3). *S. geminata*,* N. guatemalensis*, and *M. ebeninum* were identified as dominant species because they controlled a large proportion of baits at which they were present and have a high mean score abundance (Table 3). *P. fallax* and *B. patagonicus* were identified as subdominant species because they controlled a moderate proportion of baits at which they were present and have a moderate mean score abundance (Table 3). All other species were identified as subordinate species (Table 3). Maps of spatial distribution of species are provided in the Appendix (Figure A2).

**Table 3:** Occurrence of dominant, subdominant, and subordinate ants at baits

	Recorded baits (%)	Controlled baits (%)	Mean abundance score
Dominant			
*Solenopsis geminata*	20.65	43.50	4.12
*Nylanderia guatemalensis*	75.74	32.12	3.26
*Monomorium ebeninum*	25.37	28.48	3.23
Subdominant			
*Pheidole fallax*	70.00	20.10	2.87
*Brachymyrmex patagonicus*	77.04	14.90	2.74
Subordinate			
*Cardiocondyla obscurior*	66.02	2.94	2.07
*Wasmannia auropunctata*	10.09	3.67	1.93
*Odontomachus brunneus*	26.48	0	1.86
*Camponotus sexguttatus*	34.44	1.88	1.59
*Paratrechina longicornis*	11.48	0	1.38

The abundance of *S. geminata*,* M. ebeninum* was greater in the afternoon than in the morning, while the abundance *N. guatemalensis* was greater in the morning than in the afternoon (Table 4). We found no difference between the abundances at baits in the morning and in the afternoon for *P. fallax* and *B. patagonicus* (Table 4).

**Table 4:** Comparisons of the abundances in the morning and in the afternoon of dominant, subdominant, and subordinate ants at baits (Kruskal–Wallis tests)

	Mean abundance (95% CI)				
	Morning	Afternoon	χ^2^	*df*	*p*
Dominant					
*Solenopsis geminata*	4.49 (3.37–5.60)	6.85 (5.49–8.20)	6.2	1	**.013**
*Nylanderia guatemalensis*	11.92 (10.86–12.97)	9.71 (8.65–10.78)	19.1		**<.001**
*Monomorium ebeninum*	3.20 (2.31–4.09)	6.02 (4.75–7.30)	6.9	1	**.009**
Subdominant					
*Pheidole fallax*	8.81 (7.55–10.07)	8.05 (6.82–9.29)	2.6	1	.105
*Brachymyrmex patagonicus*	7.62 (6.52–8.71)	10.67 (9.23–12.11)	0.7	1	.416
Subordinate					
*Cardiocondyla obscurior*	2.23 (1.98–2.49)	2.27 (2.00–2.55)	0.4	1	.544
*Wasmannia auropunctata*	0.43 (0.27–0.58)	0.49 (0.26–0.73)	1.1	1	.285
*Odontomachus brunneus*	0.93 (0.77–1.09)	0.60 (0.46–0.74)	19.9		**<.001**
*Camponotus sexguttatus*	0.78 (0.64–0.93)	0.99 (0.74–1.24)	0.1	1	.807
*Paratrechina longicornis*	0.12 (0.09–0.16)	0.32 (0.19–0.45)	5.1	1	**.024**

### 3.3 Probability of transition matrices

**Figure 2:**
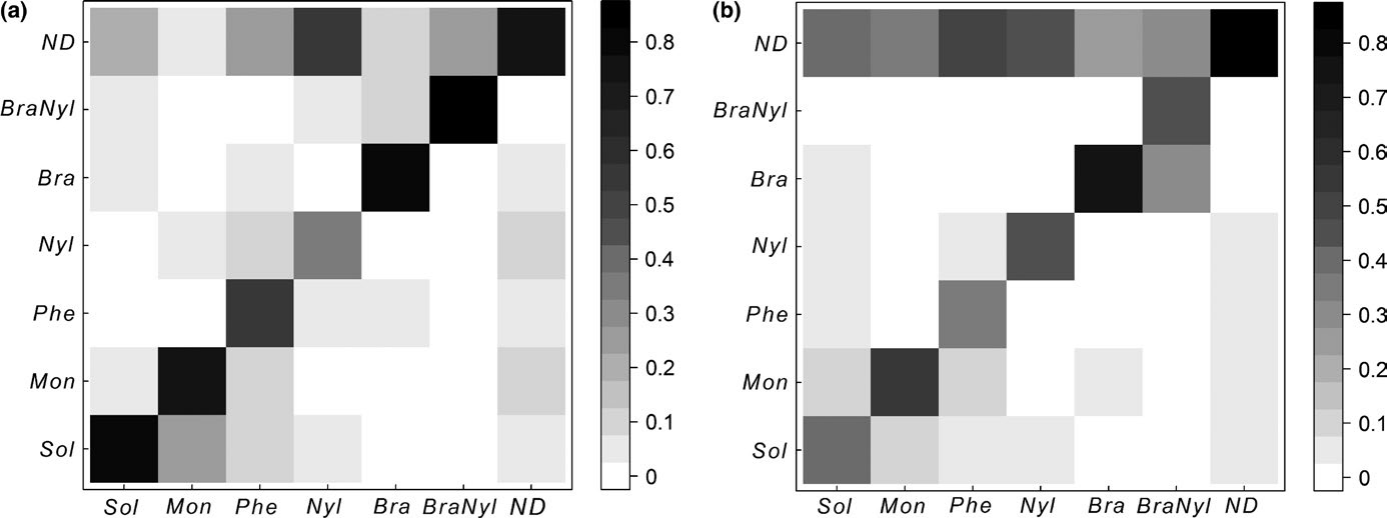
Probability of transition matrices. These figures display the probability that a numerically dominant species (listed on *X*‐axis) will maintain its numerical dominance on the resource or lose it to the benefit of another dominant or subdominant species (listed on *Y*‐axis) from: (a) sampling time 1 to sampling time 2; (b) sampling time 2 to sampling time 3. In (a) and (b), the diagonal indicates the probability that a species maintains dominance on the resource between sampling times. *Sol*,* S. geminata*;* Mon*,* M. ebeninum*;* Phe*,* P. fallax*;* Nyl*,* N. guatemalensis*;* Bra*,* B*. *patagonicus*;* BraNyl*, codominance *B. patagonicus/N. guatemalensis*;*ND*, no dominant species

Based on hierarchical classification and clustering methods, only the three dominant and the two subdominant species contributed to the community structure at the subplot scale. We characterized seven typical groups corresponding to different community structures at the subplot scale (Table A1). Five of the groups were numerically dominated by one species, that is, groups 1–5 were numerically dominated by *S. geminata*,* M. ebeninum*,* P. fallax*,* N. guatalamensis*, and *B. patagonicus*, respectively. Group 6 was numerically co‐dominated by *N. guatalamensis* and *B. patagonicus*, and group 7 was not numerically dominated by any species. The probability that a species maintained numerical dominance of a resource between sampling times 1 and 2 was high for *S. geminata*,* M. ebeninum*,* B. patagonicus*, and *N. guatalamensis* + *B. patagonicus* (Table 5, Figure 2a); these probabilities were lower for *P. fallax* and *N. guatemalensis* (Table 5, Figure 2a). The probability that a species maintained numerical dominance of a subplot resource between sampling times 2 and 3 was again high for the *B. patagonicus* group (Table 5, Figure 2b), and Markov chain simulations indicated that the probability of this group (group 5) maintaining numerical dominance between sampling times 1 and 3 was 0.6 (Table 5). For each species, most of the subplots where numerical dominance was not maintained were taken over by group 7, that is, the group that lacked a numerically dominant species (Figure 2a,b). This was particularly true for *P. fallax* and *N. guatemalensis*, two species that displayed low probabilities of maintaining numerical dominance on subplots (Figure 2a,b). We also observed that subplots categorized as group 7 (which lacked a numerically dominant species) tended to stay in the group 7 at the following sampling time (Figure 2a,b).

**Table 5:** The probability that a species maintained dominance of a subplot resource between sampling times as determined by Markov chain simulations (10,000 iterations per group). *Sol*,* Solenopsis*;* Mon*,* M. ebeninum*;* Phe*,* P. fallax*;* Nyl*,* N. guatemalensis*;* Bra*,* B. patagonicus*;* BraNyl*, codominance *B. patagonicus/N. guatemalensis*;* ND*, no dominant species

Sampling times	Probability for the indicated dominant species						
	*Sol*	*Mon*	*Phe*	*Nyl*	*Bra*	*BraNyl*	*ND*
1–2	0.78	0.70	0.53	0.32	0.79	0.82	0.72
2–3	0.40	0.57	0.33	0.47	0.76	0.46	0.57
1–3	0.32	0.40	0.18	0.16	0.60	0.38	0.40

### 3.4 Autocorrelation

**Figure 3:**
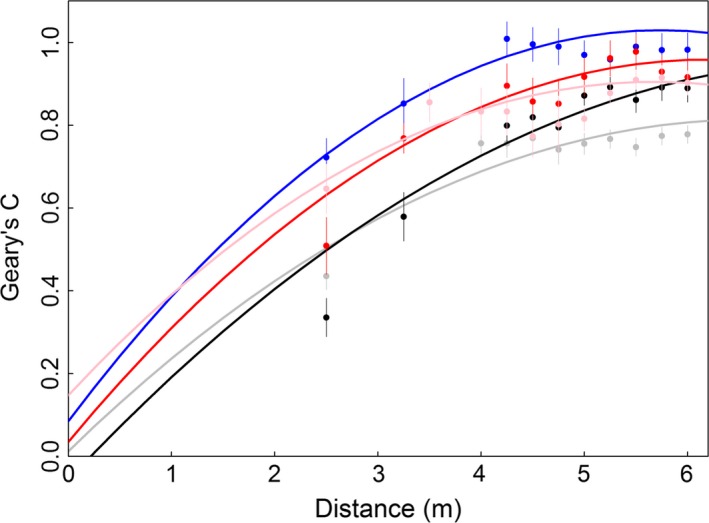
Mean values for Geary's C as affected by distance between two subplots. Points correspond to raw data, and curves are fitted from linear regression models. Gray: *N. guatemalensis* (*r* ^2^ = 0.83); black: *B. patagonicus* (*r* ^2^ = 0.94); blue: *P. fallax* (*r* ^2^ = 0.78); red: *S. geminata* (*r* ^2^ = 0.83); pink: *M. ebeninum* (*r* ^2^ = 0.38)

For the abundance of each species, we found a positive spatial autocorrelation among neighboring subplots that were separated by as much as 6.0 m, and that values for Geary's *C* (according to LMs) were positively associated with distance (Figure 3) and we considered spatial autocorrelation distances of 3 m for *P. fallax*, 4 m for *M. ebeninum* and *S. geminata*, and 5 m for *N. guatemalensis* and *B. patagonicus*. For analyses in the next section, we used these spatial autocorrelation distances to calculate the abundance of each species present in the neighborhood of *P. fallax* and *M. ebeninum*,* S. geminata* and *N. guatemalensis*, and *B. patagonicus*.

### 3.5 Subplot scale dynamics and correlations between ant species abundances

**Figure 4:**
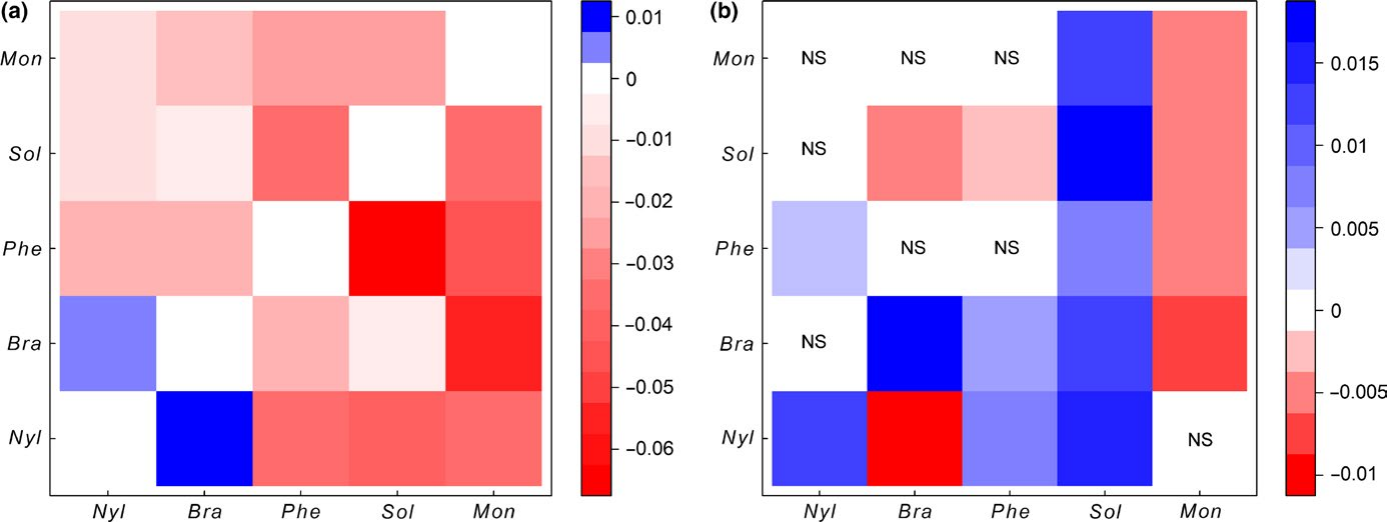
Estimates of correlations between ant species abundances. (a) Estimated effects of the abundance of a species (listed on *Y*‐axis) in subplots on the abundance of a species (listed on the *X*‐axis) in the same subplots. (b) Estimated effects of the abundance of a species (listed on the *Y*‐axis) in neighboring subplots on the abundance of a species (listed on the *X*‐axis) in subplots. *Sol*,* S. geminata*;* Mon*,* M. ebeninum*;* Phe*,* P. fallax*;* Nyl*,* N. guatemalensis*;* Bra*,* B*. *patagonicus*

Statistical analyses of the relationships between the abundance of each ant taxon (interspecific interactions) are provided in the supporting information (Tables A2‐A6), and the results are summarized in Figure 4a,b. Among the 15 pairwise local (within subplots) interspecific correlations, 14 were symmetrically negative (−/−), and one was symmetrically positive (+/+). The abundance of *S. geminata* was negatively correlated with the local abundances of other ant species (Figure 4a) but was positively correlated with the abundances of conspecifics and other ant species in its neighborhood (Table A2, Figure 4b). The abundance of *M. ebeninum* was negatively correlated with the local abundances of other ant species (Figure 4a) and with the abundances of conspecifics and other ant species in its neighborhood, except that *N. guatemalensis* abundance in the neighborhood was not correlated with *M. ebeninum* abundance (Table A3, Figure 4b). The abundance of *P. fallax* was negatively correlated with the local abundances of other ant species (Figure 4a); the abundance of *P. fallax* was also negatively correlated with the abundance of *S. geminata* in its neighborhood and was positively correlated with the abundances of *N. guatemalensis* and *B. patagonicus* in its neighborhood (Figure 4b). The abundance of *P. fallax* conspecifics and *M. ebeninum* in the neighborhood was not correlated with the abundance of *P. fallax* (Table A4, Figure 4b). The abundance of *N. guatemalensis* was negatively correlated with the local abundances of other ant species except that it was positively correlated with *B. patagonicus* abundance (Table A6, Figure 4A). The abundance of *B. patagonicus* was negatively correlated with the local abundances of other ant species except that it was positively correlated with *N. guatemalensis* abundance (Table A5, Figure 4a). The abundance of *B. patagonicus* was positively correlated with the abundances of conspecifics in its neighborhood and negatively correlated with the abundances of *S. geminata* or *N. guatemalensis* in its neighborhood (Table A5, Figure 4b). No correlation was detected between the abundance of *B. patagonicus* and the abundances of *P. fallax* or *M. ebeninum* (Table A5, Figure 4b). The abundance of *N. guatemalensis* was positively correlated with the abundances of conspecifics and *P. fallax* in its neighborhood, while the abundances of other ant species in its neighborhood were not correlated with its local abundance (Table A6, Figure 4b). The random effect of date on intercept and the random slope effect of dominant group significantly improved the GLMMs for all model species of abundance (Tables A2‐A6).

## 4 Discussion

We performed bait and pitfall trapping experiments in a very simplified and homogeneous agroecosystem for studying ant community structure. Doing so minimized the effects of habitat‐related factors that can affect community structure. The pattern analysis provided information on temporal and spatial dynamics of dominant, subdominant, and subordinate ants foraging in a single‐crop banana agroecosystem.

We found that the community of ground‐dwelling ants was dominated by *Solenopsis geminata*,* Monomorium ebeninum*,* Nylanderia guatemalensis*,* Pheidole fallax*, and *Brachymyrmex patagonicus*. Our results showed that species generally maintained numerical dominance of a subplot (bait) throughout each 180‐min sampling date. *Solenopis geminata* displayed a high probability of maintaining numerical dominance, although the probability decreased greatly with sampling time, that is, with resource consumption. This species actively recruits to food sources and is very aggressive toward competitors (Trager, 1991). When *S. geminata* did not maintain numerical dominance, it was usually not to the benefit of a particular species. A similar pattern of maintaining numerical dominance was observed for *M. ebeninum*, which is consistent with Hanson and Gauld (1995), who reported that this species behaves like *Solenopis* in the field. However, when *M. ebeninum* did not maintain numerical dominance, it was mostly to the benefit of *S. geminata*, suggesting that the two species may have similar ecological niches. This is consistent with the similarity that we observed in their diurnal and nocturnal foraging activity and with the daily variation of occurrence at baits. *Brachymyrmex patagonicus* displayed high probabilities of maintaining numerical dominance at baits, and the decrease in its probability of maintaining numerical dominance over time was lower for this species than for the other species in our study. MacGown, Hill, and Deyrup (2007) reported that *Brachymyrmex* spp. in general and *B. patagonicus* in particular have the ability to coexist with a variety of other dominant species. These authors also suggest that *B. patagonicus* may be protected by potent chemicals. *N. guatemalensis* displayed the lowest probability of maintaining numerical dominance at baits. This is consistent with LaPolla, Brady, and Shattuck (2011), who described *Nylanderia* spp. as efficient foragers that rapidly find and recruit to resources but that rarely can defend the resources against other ants that arrive later. *P. fallax* displayed low probabilities of maintaining numerical dominance at baits. Perfecto and Vandermeer (2011) found that *Pheidole subarmata* tends to lose dominance at baits against *S. geminata*. In our study, *P. fallax* was apparently kept low at baits not only by *S. geminata*, which is a strong interference competitor (Perfecto & Vandermeer, 2011), but also by all of the other dominant and subdominant ant species. *Nylanderia* spp. are known to be good exploitation competitors (LaPolla et al., 2011), but our results suggest that, depending on the context and the competitors that they face, *Nylanderia* ants may also be interference competitors.

However, the patterns we observed may also depend, at least in part, on other factors such as daily variation in foraging activity because of thermal constraints. Thermal constraints have been shown to disrupt hierarchies in ant communities (Bestelmeyer, 2000; Cerdá, Retana, & Cros, 1997). We found that two of the three dominant species (*M. ebeninum* and *S. geminata*) were more abundant at baits in the afternoon than in the morning while the other one (*N. guatemalensis*) displayed the opposite trends. Moreover, the foraging activity of two former species was higher the day than at night, while it was the opposite for *N. guatemalensis*. The subdominant ants (*P. fallax* and *B. patagonicus*) had greater foraging activity during the day, with no difference in abundance at baits between the afternoon and the morning, while being the two most abundant species at night. Overall, the variation in foraging activities may explain the coexistence of dominant, subdominant, and subordinates species in this agroeocosystem limited in habitat structure and diversity because of the induction of temporal niche partitioning (Albrecht & Gotelli, 2001; Cerdá, Retana, & Manzaneda, 1998).

All of the coefficients of local (i.e., at baits) interspecific correlations, with the exception of one pairwise correlation, were symmetrically negative, suggesting that interference competition prevails in this community of ground‐dwelling ants. The coefficients of local interactions, however, were symmetrically positive between *B. patagonicus* and *N. guatemalensis*. Thus, competitive exclusion at an ephemeral food source does not occur between these two species of Formicinae, which seem to tolerate each other.

The positive correlation between the local abundance and neighborhood abundance of a considered species may reflect the absence of intraspecific competition between colonies. Indeed, *S. geminata* is known to be, at least temporarily, polygynous (Trager, 1991). Some *Nylanderia* spp. may also be polygynous (Arcila, Ulloa‐Chacon, & Gomez, 2002), and MacGown et al. (2007) reported that *B. patagonicus* colonies may be situated very close to each other, displaying considerable mutual tolerance. McGlynn (2010) demonstrated that polygyny increases in response to the density of ant competition. The positive correlation between the local abundance of an ant species with the neighborhood abundance of another species may reflect an overlap in spatial distribution, which seems to be the usual pattern in tropical ant communities (Soares & Schoereder 2001). This should be particularly true in homogeneous agroecosystems where food and nesting resources display very few variations. Here, we found positive correlations between the local abundance of *P. fallax* and the neighborhood abundance of *N. guatemelensis*, and between the local abundance of *N. guatemelensis* and the neighborhood abundance of *P. fallax*. Similar spatial associations have been reported between unidentified species in the genus *Pheidole* and *Paratrechina* (Chong, Hoffmann, & Thomson, 2011), the latter genus having been recently separated into the genera *Paratrechina* and *Nylanderia* (LaPolla, Brady, & Shattuck, 2010). This positive spatial association, despite the competitive interference found between these two species at baits, could result from the dissimilarity between their temporal foraging activity. Indeed, our data support this hypothesis as *P. fallax* foraging activity is greater during the day while the foraging activity of *N. guatemalensis* greater at night. This should lead to the temporal sharing of resources. The positive spatial association could also result from a similarity in foraging behavior. We found the lowest probabilities of maintaining dominance in these two species, both of which are good exploitative competitors that rapidly discover resources (Itzkowitz & Haley, 1983; LaPolla et al., 2011), had a similar probability to losing dominance at baits to the benefit of the other. This should lead to the spatial sharing of resources at such fine spatial scale. We also found a positive correlation between the local abundance of *P. fallax* and the neighborhood abundance of *B. patagonicus*, whose local abundance was not correlated with the neighborhood abundance of *P. fallax*. Because *B. patagonicus* was highly numerically dominant at baits and had a high probability of maintaining its numerical dominance at baits, it is not surprising that the presence of the *P. fallax* in the neighborhood did not apparently affect *B. patagonicus*.

To our knowledge, this is the first study reporting information on ant community in a banana crop agrosystem in Martinique. In summary, we found that three dominant and two subdominant species structure the ant community in a very simplified banana agrosystem. All these species generally maintain numerical dominance at a “momentary” resource and that interference competition probably prevails in this ground‐dwelling community. However, temporal and spatial niche partitioning may also explain, at least partly, the observed pattern. Moreover, the presence of invasive species, such as the fire ant *S. geminata*, the rover ant *B. patagonicus*, the little fire ant *W. auropunctata*, and the crazy ant *P. longicornis*, might greatly affect ecological processes and ecosystems (Lach & Hooper‐Bui, 2010). Thus, we cannot extent our results to other ecosystems and consider that further studies in very simplified agrosystems should be carried out to better assess the relative contribution of these ecological processes in the structuring of ant communities.

## Appendix 1

### 1.1

**Table A1:** The mean composition of the seven typical groups of ants that differed in community structure in subplots of a banana field. The values indicate the mean (SE) number of individuals per subplot and sampling time of each species per group. The emboldened values indicate the dominant species in each group. Groups 1–5 had one dominant species; group 6 had two dominant species; and group 7 had no dominant species

Species	Group 1	Group 2	Group 3	Group 4	Group 5	Group 6	Group 7
*Nylanderia guatemalensis*	3.72 (0.66)	5.41 (0.84)	4.19 (0.51)	**34.91 (1.10)**	6.06 (0.90)	**25.09 (2.14)**	9.42 (0.39)
*Brachymyrmex patagonicus*	6.57 (1.21)	2.47 (0.31)	2.58 (0.48)	5.82 (0.92)	**46.06 (0.82)**	**46.36 (1.22)**	4.45 (0.28)
*Pheidole fallax*	2.83 (0.46)	3.16 (0.64)	**44.14 (0.84)**	2.70 (0.39)	2.44 (0.61)	3.42 (1.52)	4.28 (0.30)
*Solenopsis geminata*	**46.79 (0.77)**	1.76 (0.88)	0.38 (0.31)	1.42 (0.13)	0.68 (0.72)	0.06 (0.06)	1.41 (0.22)
*Monomorium ebeninum*	2.5 (0.65)	**48.0 (0.60)**	0.98 (0.24)	0.20 (0.06)	0.04 (0.03)	0.0 (0.0)	1.37 (0.22)

**Table A2:** Selection of the model explaining the local abundance of *Solenopsis geminata*. Results of likelihood ratio tests are also shown for the significance of retaining fixed and random effects in the model. Fixed or random effects were kept in the selected model if the *p*‐value was ≤.05

Model	*df*	Δ*df*	AIC	ΔAIC	Log‐likelihood	χ^2^	*p*‐value
Selected	13		8,072.8		−4,023.4		
Fixed effects							
Sampling time	12	1	8,242.7	169.9	−4,109.3	171.8	<.0001
Local abundance of							
*Pheidole fallax*	12	1	9,986.4	1,913.6	−4,981.2	1,915.6	<.0001
*Monomorium ebeninum*	12	1	8,647.9	575.1	−4,282.1	517.3	<.0001
*Brachymyrmex patagonicus*	12	1	8,086.1	13.3	−4,031	15.3	<.0001
*Nylanderia guatemalensis*	12	1	8,511.8	439	−4,243.9	441.0	<.0001
Neighboring abundance of							
*P. fallax*	12	1	8,079.9	7.1	−4,027.9	9.1	.003
*Solenopsis geminata*	12	1	8,239.5	166.7	−4,107.8	168.7	<.0001
*B. patagonicus*	12	1	8,116.8	44	−4,046.4	46.0	<.0001
*M. ebeninum*	12	1	8,152.7	79.9	−4,064.4	81.9	<.0001
*N. guatemalensis*	12	1	8,128.6	55.8	−4,052.3	57.8	<.0001
Random effects							
Date	12	1	8,160	87.2	−4,068	89.2	<.0001
Dominant group	12	1	19,108.9	11,036.1	−9,542.5	11,038	<.0001

**Table A3:** Selection of the model explaining the local abundance of *Monomorium ebeninum*. Results of likelihood ratio tests are also shown for the significance of retaining fixed and random effects in the model. Fixed or random effects were kept in the selected model if the *p*‐value was ≤.05

Model	*df*	Δ*df*	AIC	ΔAIC	Log‐likelihood	χ^2^	*p*‐value
Selected	12		8,970.8		−4,473.4		
Fixed effects							
Sampling time	11	1	9,005.8	35	−4,491.9	37.0	<.0001
Local abundance of							
*Pheidole fallax*	11	1	9,971.1	1,000.3	−4,974.6	1,002.3	<.0001
*Solenopsis geminata*	11	1	10,028.3	1,057.5	−5,003.1	1,059.5	<.0001
*Brachymyrmex patagonicus*	11	1	9,460.3	489.5	−4,719.2	491.5	<.0001
*Nylanderia guatemalensis*	11	1	9,320	349.2	−4,649	351.2	<.0001
Neighboring abundance of							
*P. fallax*	11	1	8,976.8	6	−4,477.4	8.0	.005
*S. geminata*	11	1	8,975.9	5.1	−4,476.9	7.1	.008
*B. patagonicus*	11	1	8,976	5.2	−4,477	7.2	.007
*Monomorium ebeninum*	11	1	8,981.5	10.7	−4,479.8	12.7	.0004
*N. guatemalensis*	–	–	–	–	–	–	.09
Random effects							
Date	11	1	9,017.3	46.5	−4,497.6	48.5	<.0001
Dominant group	11	1	16,169.8	7,199	−8,073.9	7,200.9	<.0001

**Table A4:** Selection of the model explaining the local abundance of *Pheidole fallax*. Results of likelihood ratio tests are also shown for the significance of retaining fixed and random effects in the model. Fixed or random effects were kept in the selected model if the *p*‐value was ≤.05

Model	*df*	Δ*df*	AIC	Log‐likelihood	χ^2^	*p*‐value
Selected	11		12,215		−6,096.7		
Fixed effects							
Sampling time	10	1	12,264	49	−6,122.3	51.2	<.0001
Local abundance of							
*Solenopsis geminata*	10	1	13,574	1,359	−6,777	1,360.6	<.0001
*Monomorium ebeninum*	10	1	12,670	455	−6,324.7	456.2	<.0001
*Brachymyrmex patagonicus*	10	1	12,517	302	−6,248.3	303.2	<.0001
*Nylanderia guatemalensis*	10	1	13,195	980	−6,587.4	981.4	<.0001
Neighboring abundance of							
*Pheidole fallax*	–	–	–	–	–	–	.079
*S. geminata*	10	1	12,218	3	−6,098.8	4.2	.039
*B. patagonicus*	10	1	12,229	14	−6,104.6	15.8	<.0001
*M. ebeninum*	–	–	–	–	–	–	.525
*N. guatemalensis*	10	1	12,233	18	−6,106.7	20.1	<.0001
Random effects							
Date	10	1	12,298	83	−6,138.9	84.4	<.0001
Dominant group	10	1	16,391	4,176	−8,185.6	4,177.8	<.0001

**Table A5:** Selection of the model explaining the local abundance of *Brachymyrmex patagonicus*. Results of likelihood ratio tests are also shown for the significance of retaining fixed and random effects in the model. Fixed or random effects were kept in the selected model if the *p*‐value was ≤.05

Model	*df*	Δ*df*	AIC	ΔAIC	Log‐likelihood	χ^2^	*p*‐value
Selected	11		10,714		−5,346.2		
Fixed effects							
Sampling time	10	1	10,793	79	−5,386.4	80.3	<.0001
Local abundance of							
*Pheidole fallax*	10	1	11,069	355	−5,524.3	356.2	<.0001
*Solenopsis geminata*	10	1	10,742	28	−5,361.1	29.6	<.0001
*Monomorium ebeninum*	10	1	10,775	61	−5,377.1	62.1	<.0001
*Nylanderia guatemalensis*	10	1	10,734	20	−5,357.2	21.9	<.0001
Neighboring abundance of							
*P. fallax*	–	–	–	–	–	–	.15
*S. geminata*	10	1	10,802	88	−5,391.2	89.9	<.0001
*Brachymyrmex patagonicus*	10	1	10,950	236	−5,465.2	237.8	<.0001
*M. ebeninum*	–	–	–	–	–	–	.34
*N. guatemalensis*	10	1	10,723	9	−5,351.5	10.5	.09
Random effects							
Date	10	1	11,510	796	−5,745	797.5	<.0001
Dominant group	10	1	16,429	5,715	−8,204.3	5,716.2	<.0001

**Table A6:** Selection of the model explaining the local abundance of *Nylanderia guatemalensis*. Results of likelihood ratio tests are also shown for the significance of retaining fixed and random effects in the model. Fixed or random effects were kept in the selected model if the *p*‐value was ≤.05

Model	*df*	Δ*df*	AIC	ΔAIC	Log‐likelihood	χ^2^	*p*‐value
Selected	10		10,277		−5,128.3		
Fixed effects							
Sampling time	9	1	10,300	23	−5,141.2	25.9	<.0001
Local abundance of							
*Pheidole fallax*	9	1	10,815	538	−5,398.5	540.4	<.0001
*Solenopsis geminata*	9	1	10,422	145	−5,201.9	147.3	<.0001
*Monomorium ebeninum*	9	1	10,371	94	−5,176.7	96.8	<.0001
*Brachymyrmex patagonicus*	9	1	10,310	33	−5,145.8	35.0	<.0001
Neighboring abundance of							
*P. fallax*	9	1	10,295	18	−5,138.5	20.4	<.0001
*S. geminata*	–	–	–	–	–	–	.16
*B. patagonicus*	–	–	–	–	–	–	.50
*M. ebeninum*	–	–	–	–	–	–	.71
*Nylanderia guatemalensis*	9	1	10,468	191	−5,225.1	193.58	<.0001
Random effects							
Date	9	1	11,941	1,664	−5,961.5	1,666.5	<.0001
Dominant group	9	1	12,332	2,055	−6,157	2,057.4	<.0001
